# Somatic Populations of PGT135–137 HIV-1-Neutralizing Antibodies Identified by 454 Pyrosequencing and Bioinformatics

**DOI:** 10.3389/fmicb.2012.00315

**Published:** 2012-09-11

**Authors:** Jiang Zhu, Sijy O’Dell, Gilad Ofek, Marie Pancera, Xueling Wu, Baoshan Zhang, Zhenhai Zhang, NISC Comparative Sequencing Program, James C. Mullikin, Melissa Simek, Dennis R. Burton, Wayne C. Koff, Lawrence Shapiro, John R. Mascola, Peter D. Kwong

**Affiliations:** ^1^Vaccine Research Center, National Institute of Allergy and Infectious Diseases, National Institutes of HealthBethesda, MD, USA; ^2^Department of Biochemistry and Molecular Biophysics, Columbia UniversityNew York, NY, USA; ^3^NIH Intramural Sequencing Center, National Human Genome Research Institute, National Institutes of HealthBethesda, MD, USA; ^4^International AIDS Vaccine InitiativeNew York, NY, USA; ^5^Department of Immunology and Microbial Science, The Scripps Research InstituteLa Jolla, CA, USA; ^6^International AIDS Vaccine Initiative Neutralizing Antibody Center, The Scripps Research InstituteLa Jolla, CA, USA; ^7^Ragon Institute of MGH, MIT, and HarvardCambridge, MA, USA

**Keywords:** antibody bioinformatics, high-throughput sequencing, HIV-1, immunity, N-linked glycan

## Abstract

Select HIV-1-infected individuals develop sera capable of neutralizing diverse viral strains. The molecular basis of this neutralization is currently being deciphered by the isolation of HIV-1-neutralizing antibodies. In one infected donor, three neutralizing antibodies, PGT135–137, were identified by assessment of neutralization from individually sorted B cells and found to recognize an epitope containing an N-linked glycan at residue 332 on HIV-1 gp120. Here we use next-generation sequencing and bioinformatics methods to interrogate the B cell record of this donor to gain a more complete understanding of the humoral immune response. PGT135–137-gene family specific primers were used to amplify heavy-chain and light-chain variable-domain sequences. Pyrosequencing produced 141,298 heavy-chain sequences of IGHV4-39 origin and 87,229 light-chain sequences of IGKV3-15 origin. A number of heavy and light-chain sequences of ∼90% identity to PGT137, several to PGT136, and none of high identity to PGT135 were identified. After expansion of these sequences to include close phylogenetic relatives, a total of 202 heavy-chain sequences and 72 light-chain sequences were identified. These sequences were clustered into populations of 95% identity comprising 15 for heavy chain and 10 for light chain, and a select sequence from each population was synthesized and reconstituted with a PGT137-partner chain. Reconstituted antibodies showed varied neutralization phenotypes for HIV-1 clade A and D isolates. Sequence diversity of the antibody population represented by these tested sequences was notably higher than observed with a 454 pyrosequencing-control analysis on 10 antibodies of defined sequence, suggesting that this diversity results primarily from somatic maturation. Our results thus provide an example of how pathogens like HIV-1 are opposed by a varied humoral immune response, derived from intrinsic mechanisms of antibody development, and embodied by somatic populations of diverse antibodies.

## Introduction

Recent years have seen revolutions in both genomics and computational science (Lander et al., 2001; Venter et al., 2001; Chen et al., 2012). In both of these fields, capabilities are advancing exponentially (Kahn, 2011). The impact of this non-linear development on biology is pervasive and multifaceted. With respect to virus research, the influence has been profound and is the focus of this special issue of *Frontiers*. Medical interest in viruses is focused on pathogens and their infection, and the biological mirror of infection is the host immune response. Advances in genomics and computational science have the potential for an equally profound impact on our understanding of the immune response. Here we focus on the application of new genomic and computational techniques, particularly 454 pyrosequencing of B cell transcripts (Reddy et al., 2009; Reddy and Georgiou, 2011; Wu et al., 2011) and systems-level bioinformatics (Kitano, 2002), to understand the antibody response to infection.

The human immunodeficiency virus type I, HIV-1, is the etiological agent of a global pandemic, which has killed over 30 million people, and currently infects ∼1% of adults worldwide (UNAIDS, 2010). HIV-1 is a retrovirus and member of the lentivirus genus (Gonda et al., 1985; Sonigo et al., 1985). Global genetic diversity of HIV-1 is extraordinarily high (Starcich et al., 1986; Korber et al., 2001), and this is thought to result from the low fidelity of its genome replication (Preston et al., 1988) as well as the persistent nature of the infection: the diversity of HIV-1 virus within a single individual after 6 years of infection is equivalent to the global diversity of H1N1 influenza observed annually (Korber et al., 2001). Infection by HIV-1 elicits many antibodies, but in general these are not capable of neutralization of diverse strains of HIV-1. However, after several years of infection, 10–25% of infected individuals develop broadly neutralizing antibodies (Li et al., 2007; Gray et al., 2009; Sather et al., 2009; Simek et al., 2009; Stamatatos et al., 2009; Doria-Rose et al., 2010; Gnanakaran et al., 2010). These antibodies provide little or no benefit to the infected host, as the evolution of the virus outpaces the immune response (Parren et al., 1999; Poignard et al., 1999; Wei et al., 2003). Nevertheless these antibodies, when tested in humanized mice or macaque models by passive antibody transfer, impart effective immunity to challenge with HIV-1 or simian/human chimeric immunodeficiency viruses (Mascola et al., 1999, 2000; Parren et al., 2001; Mascola, 2003; Veazey et al., 2003; Hessell et al., 2009a,b; Balazs et al., 2011), indicating the potential for their use as targets for re-elicitation by rationally designed vaccines (reviewed in Walker and Burton, 2010; Kwong et al., 2011). Thus, substantial interest has focused on understanding human antibodies that effectively neutralize diverse strains of HIV-1.

A number of techniques have recently been applied to identification of such antibodies. These methods – including antigen-specific B cell sorting (Scheid et al., 2009; Wu et al., 2010) and direct assessment of neutralization by antibodies secreted from individually sorted B cells (Walker et al., 2009, 2011), each coupled to single B cell sequencing techniques – have so far yielded dozens of broadly HIV-1-neutralizing antibodies. These antibodies represent an extraordinarily sparse sampling of the humoral immune response, which typically generates roughly a billion new B cells in a healthy individual each day. We therefore asked whether the revolutionary new capabilities of next-generation sequencing (Mardis, 2008a,b; Boyd et al., 2010; Hawkins et al., 2010) and computational science could expand this sampling to generate a more complete understanding of the humoral immune response. In principle, memory B cells contain a persistent record of the antibody response to infection. As memory B cells are readily attained from blood, they provide a convenient means to access the antibody record, with B cell transcripts in peripheral blood mononuclear cells (PBMCs) providing a genetic representation. Using three antibodies, PGT135–137 from Protocol G donor 39 (Walker et al., 2011) as an example, we used 454 pyrosequencing of PCR-amplified heavy- and light-chain transcripts to capture a more comprehensive genetic record. We used bioinformatics approaches to interrogate this record, to identify populations of neutralizing antibodies, and to characterize their ontogenies. We link these ontogenies to the natural mechanisms of B cell development to provide a view of how somatic populations of antibodies engender a diverse immunological response to infection.

## Materials and Methods

### Human specimens

The PBMCs of the HIV-1 infected donor 39 were obtained from the International AIDS Vaccine Initiative (IAVI) protocol G. The same sample was used to isolate broadly neutralizing antibodies PGT135–137 (Walker et al., 2011). Human peripheral blood samples were collected after obtaining informed consent and appropriate Institutional Review Board (IRB) approval.

### Sample preparation for 454 pyrosequencing

Ten previously described heavy-chain plasmids with known sequences (Wu et al., 2011) were selected to assess 454 pyrosequencing error. Ten plasmids (100 ng each) were combined in 35 μl water, and 1 μl of the ten-plasmid combination was used to template polymerase chain reactions (PCRs). The heavy and kappa chain PCR samples for 454 pyrosequencing from donor 39 were prepared as described (Wu et al., 2011) with minor modifications. Briefly, mRNA was extracted from 20 million PBMCs into 200 μl of elution buffer (Oligotex kit, Qiagen), then concentrated to 10–30 μl by centrifuging the buffer through a 30 kD micron filter (Millipore). The reverse transcription was performed in one or multiple 35 μl-reactions, each composed of 13 μl of mRNA, 3 μl of oligo(dT)_12–18_ at 0.5 μg/μl (Invitrogen), 7 μl of 5× first strand buffer (Invitrogen), 3 μl of RNase Out (Invitrogen), 3 μl of 0.1 M DTT (Invitrogen), 3 μl of dNTP mix (each at 10 mM), and 3 μl of SuperScript II (Invitrogen). The reactions were incubated at 42°C for 2 h. The cDNAs from each reaction were combined, applied to the NucleoSpin Extract II kit (Clontech), and eluted in 20 μl of elution buffer. In this way, 1 μl of the cDNA comprised transcripts from 1 million PBMCs. The immunoglobulin gene family-specific PCR was set up in a total volume of 50 μl, using 1 μl of the heavy-chain plasmid mix or 5 μl of the cDNA as template (equivalent of transcripts from 5 million PBMCs). The DNA polymerase systems used was the Platinum *Taq* High-Fidelity (HiFi) DNA Polymerase System (Invitrogen). According to the instructions of the manufacturer, the reaction mix was composed of water, 5 μl of 10× buffer, and 1 μl of supplied MgSO4, 2 μl of dNTP mix (each at 10 mM), 1–2 μl of primers (Table S1 in Supplementary Material) at 25 μM, and 1 μl of Platinum Taq HiFi DNA polymerase. The primers each contained the appropriate adaptor sequences (XLR-A or XLR-B) for subsequent 454 pyrosequencing. The PCRs were initiated at 95°C for 30 s, followed by 25 cycles of 95°C for 30 s, 58°C for 30 s, and 72°C for 1 min, then incubated at 72°C for 10 min. The PCR products at the expected size (∼500 bp) were gel extracted and purified (Qiagen), followed by further phenol/chloroform purification.

### 454 pyrosequencing and library preparation

The 454 pyrosequencing was carried out as described previously (Wu et al., 2011). Briefly, PCR products were quantified using Qubit (Life Technologies, Carlsbad, CA, USA). Library concentrations were determined using the KAPA Biosystems qPCR system (Woburn, MA, USA) with 454 pyrosequencing standards provided in the KAPA system. Pyrosequencing of the PCR products was performed on a GS FLX sequencing instrument (Roche-454 Life Sciences, Bradford, CT, USA) using the manufacturer’s suggested methods and reagents. Initial image collection was performed on the GS FLX instrument and subsequent signal processing, quality filtering, and generation of nucleotide sequence and quality scores were performed on an off-instrument linux cluster using 454 application software (version 2.5.3). The amplicon quality filtering parameters were adjusted based on the manufacturer’s recommendations (Roche-454 Life Sciences Application Brief No. 001-2010). Quality scores were assigned to each nucleotide using methodologies incorporated into the 454 application software to convert flowgram intensity values to Phred-based quality scores and as described (Brockman et al., 2008). The quality of each run was assessed by analysis of internal control sequences included in the 454 pyrosequencing reagents. Reports were generated for each region of the PicoTiterPlate (PTP) for both the internal controls and the samples.

### Bioinformatics analysis of 454 pyrosequencing-determined antibody sequences

Our previously described bioinformatics pipeline (Wu et al., 2011) was refined and currently consists of five steps. Starting from a 454 pyrosequencing-determined antibodyome, each sequence read was (1) reformatted and labeled with a unique index number; (2) assigned to variable (*V*), diverse (*D*), and joining (*J*) gene families and alleles using an in-house implementation of IgBLAST^1^, and sequences with *E*-value > 10^−3^ for V gene assignment were rejected; (3) subjected to a template-based error-correction procedure, in which 454 pyrosequencing homopolymer errors in V, D, and J regions were detected based on the alignment to their respective germline sequences. Note that only insertion and deletion errors of less than three nucleotides were corrected. D and J gene were corrected only when their gene assignment was reliable, indicated by *E*-value < 10^−3^; (4) compared with the a set of template antibody sequences at both nucleotide level and amino-acid level using a global alignment module in CLUSTALW2 (Larkin et al., 2007); (5) subjected to a multiple sequence alignment (MSA)-based scheme to determine the third complementarity-determining region (CDR H3 or L3), which was further compared with a set of template CDR H3 or L3 sequences at nucleotide level, and to determine the sequence boundary of variable domain. For a large population of highly similar sequences, a “divide-and-conquer” procedure could be used to derive a consensus sequence to represent the population and to reduce random sequencing errors. First, a clustering using BLASTClust (Altschul et al., 1997) with a 95% sequence identity cutoff is performed on the sequence population. Then, the largest cluster is divided into 10–50 sets, for each of which a consensus can be derived from MSA. A final consensus is obtained by averaging over the subset consensuses.

Intra-donor phylogenetic analysis use the same procedure as cross-donor phylogenetic analysis, which has been described in detail in previous study (Wu et al., 2011), except that the template antibodies are from the same donor (intra-donor) rather than added exogenously (cross-donor), and intra-donor phylogenetic analysis is equally applicable to heavy and light chains. Briefly, the computational procedure consists of an iterative analysis based on the neighbor-joining (NJ) method (Kuhner and Felsenstein, 1994) implemented in CLUSTALW2 (Larkin et al., 2007) and a final analysis based on the maximum-likelihood (ML) method with molecular clock implemented in DNAMLK^2^ in the PHYLIP package v3.69^3^. In the NJ-based analysis, donor sequences of a particular germline origin were first randomly shuffled and divided into subsets of no more than 5,000 sequences. Then, PGT135–137 and respective germline sequence, IGHV4-39*07 for heavy chain and IGKV3-15*01 for light chain, were added to each subset. A NJ tree was constructed for each subset using the “Phylogenetic trees” option in CLUSTALW2 (Larkin et al., 2007). The donor sequences that clustered in the smallest branch that contains PGT135–137 were extracted from each NJ tree and combined into a new data set for the next round of analysis. The analysis was repeated until convergence, where all the donor sequences resided within a subtree containing PGT135–137 and no other sequences resided between this subtree and the root, and where further repeat of the analysis did not change the NJ tree. The ML-based analysis was used to confirm the intra-donor dendrogram derived from the NJ-based analysis. Starting from the data set obtained from the last iteration of NJ analysis, the MSA generated by CLUSTALW2 (Larkin et al., 2007) was provided as input to construct a phylogenetic tree using DNAMLK. Usually, any sequences outside the ML-defined subtree were discarded, but in this study we tested light chains identified by NJ method but immediately outside the rooted ML-defined PGT135–137 subtree. The displayed phylogenetic trees were generated using Dendroscope (Huson et al., 2007), ordered to ladderize right and rooted at the germline genes.

A description of the antibodyomics software (Antibodyomics1.0) utilized in this paper is being prepared for publication.

### Antibody expression and purification

Antibody production followed previously described procedures (Wu et al., 2011). Briefly, sequences were selected using the respective bioinformatics procedure and checked for sequencing errors using an automatic error-correction procedure followed by manual inspection. The corrected antibody sequences were synthesized (GenScript USA Inc. and Blue Heron Biotech, LLC.) and cloned into the CMV/R expression vector (Barouch and Nabel, 2005) containing the constant regions of IgG1. All synthesized heavy chains were paired with PGT137 light-chain DNA, and synthesized light chains were paired with PGT137 heavy-chain DNA for transfection. Full-length IgGs were expressed from transient transfection of 293F cells and purified using a recombinant protein-A column (Pierce).

### HIV-1 neutralization

Neutralization was measured using HIV-1 Env-pseudoviruses to infect TZM-bl cells as described (Li et al., 2005; Wu et al., 2009; Seaman et al., 2010). Neutralization curves were fit by non-linear regression using a five-parameter hill slope equation as described (Seaman et al., 2010). The 50% and 80% inhibitory concentrations (IC_50_ and IC_80_) were reported as the antibody concentrations required to inhibit infection by 50% and 80% respectively.

## Results

Experiments involving both sequencing technologies and computational analyses are described. Because variable region transcripts of antibodies are over 300 nucleotides in length and because the high similarity between different antibody transcripts precludes assembly of full sequences from fragments, we used 454 pyrosequencing, which is currently one of the few next-generation sequencing technologies to provide reads of sufficient length (Reddy et al., 2009; Reddy and Georgiou, 2011; Wu et al., 2011). However, 454 pyrosequencing is known to suffer from high error rates (Prabakaran et al., 2011). We therefore begin by characterizing the accuracy of 454 pyrosequencing applied to a set of plasmid standards consisting of known HIV-neutralizing antibodies. We then describe 454 pyrosequencing of antibody heavy-chain transcripts from donor 39 (Walker et al., 2011), and analyze these data bioinformatically and functionally. We follow this with a similar analysis of donor 39 light-chain transcripts.

### Characterization of 454 pyrosequencing errors on antibody transcripts

To investigate the extent of 454 pyrosequencing errors on the antibodyome analysis, we carried out a sequencing experiment on the heavy chains of 10 selected antibodies (Wu et al., 2011), including five from B cell sorting-based isolation, VRC01, VRC03, VRC-PG04, VRC-CH31, and VRC-CH33, one codon-optimized version of inferred reverted unmutated ancestor of VRC-PG04 (termed VRC-PG04_cog_), and four identified from previous 454 pyrosequencing study, gVRC-H3_d74_, gVRC-H6_d74_, gVRC-H12_d74_, and gVRC-H15_d74_. The plasmid sequencing data was processed with the same bioinformatics pipeline used for donor sequencing data (Figure S1 in Supplementary Material). Sequence reads were subjected to an error-correction procedure, which was aimed to fix deletion and insertion errors that cause protein translation problems (see Materials and Methods). Results obtained with and without error correction were compared to examine the effect of error correction on observed sequence variation.

**Figure 1:**
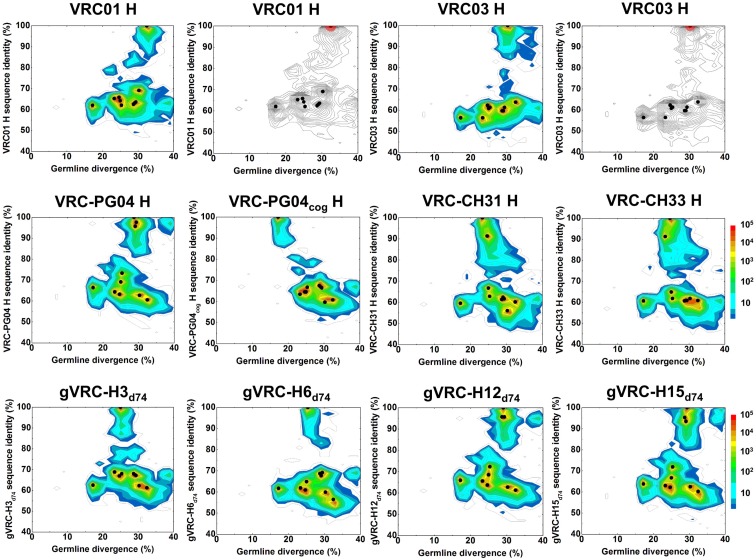
**Sequence variation as a consequence of 454 pyrosequencing for ten plasmid-control antibodies**. To quantify sequencing error, ten antibodies, input as purified plasmid DNA, were subjected to 454 pyrosequencing. Tested plasmid antibodies included VRC01, VRC03, VRC-PG04, VRC-CH31, VRC-CH33, a codon-optimized version of inferred, reverted unmutated ancestor of VRC-PG04 (termed VRC-PG04_cog_), gVRC-H3_d74_, gVRC-H6_d74_, gVRC-H12_d74_, and gVRC-H15_d74_. Heavy chain sequences are plotted as a function of sequence identity to the plasmid antibody (vertical axes) and of sequence divergence from their germline gene allele, IGHV1-2*02 (horizontal axes). The sequencing data used for divergence/identity analysis was processed by the standard bioinformatics pipeline without the error-correction step. Color coding indicates the number of sequences. For VRC01 and VRC03, additional contour plots displaying the estimated mutational error range (one root-mean-square deviation, 1.38% for VRC01 group and 1.26% for VRC03 group) have been shaded red around the input antibody.

A divergence/identity analysis was first carried out on the 10 plasmid data set, obtained without (Figure 1) and with error correction (Figure S2 in Supplementary Material). Since divergence and identity were calculated at the nucleotide level, error correction appeared to have little effect on the sequence distribution. Ideally, if the 454 pyrosequencing did not produce any errors, especially mutations, the distribution – irrespective of the antibody being used as template – would yield, on divergence/identity plots, 10 discrete points, each corresponding to one of the input sequences. In contrast, divergence/identity plots revealed broad islands centered around each of these 10 antibody sequences (Figure 1). The shape and area of each island provide a visual representation of the extent of the 454 pyrosequencing errors. As shown in Table 1, 5 of the 10 antibodies – those with an identity gap of 25% or greater to the next most closely related sequence – were easily distinguished from each other, while other more closely related variants, e.g., VRC-CH31 and VRC-CH33, overlapped (Figure 1). Based on identity considerations (Table 1) and the scope of each island in divergence/identity plots (Figure 1), a single cutoff of 75% was applied to group 454 pyrosequencing-determined sequences for VRC01, VRC03, VRC-PG04_cog_, gVRC-H3_d74_, and gVRC-H6_d74_.

**Table 1:** **Percent sequence-identity matrix of 10 plasmid antibody heavy-chain variable domains tested by 454 pyrosequencing**.

	VRC01	VRC03	VRC-PG04	VRC-CH31	VRC-CH33	VRC-PG04_cog_	gVRC-H3_d74_	gVRC-H6_d74_	gVRC-H12_d74_	gVRC-H15_d74_
VRC01	100.0	63.8	60.5	60.1	60.8	60.7	61.2	56.4	61.3	60.2
VRC03	69.1	100.0	62.7	56.0	61.8	59.6	62.2	59.9	62.7	62.5
VRC-PG04	62.5	59.7	100.0	61.3	60.6	67.5	68.0	68.7	95.7	95.3
VRC-CH31	65.3	56.4	64.3	100.0	91.3	63.4	68.8	61.9	65.6	63.0
VRC-CH33	65.8	62.3	63.2	91.3	100.0	64.5	66.9	60.9	63.7	62.2
VRC-PG04_cog_	62.0	56.4	66.4	59.5	60.6	100.0	62.5	61.7	65.9	63.8
gVRC-H3_d74_	64.2	60.8	69.1	66.7	64.9	64.5	100.0	64.9	68.5	66.7
gVRC-H6_d74_	62.0	61.3	73.3	62.8	61.8	66.7	68.0	100.0	72.3	71.9
gVRC-H12_d74_	63.1	59.7	95.7	62.3	60.8	66.9	67.5	67.7	100.0	93.5
gVRC-H15_d74_	63.6	61.3	97.6	61.6	60.8	66.4	67.2	69.2	95.7	100.0

*The heavy-chain variable domains of 10 antibodies were sequenced by 454 pyrosequencing, including VRC01, VRC03, VRC-PG04, VRC-CH31, VRC-CH33, a codon-optimized version of inferred reverted unmutated ancestor of VRC-PG04 (termed VRC-PG04_cog_), and four neutralizing antibodies whose heavy-chain variable domains were identified from donor 74 in a previous study (Wu et al., 2011) – gVRC-H3_d74_, gVRC-H6_d74_, gVRC-H12_d74_, and gVRC-H15_d74_. The diagonal values, circled and all 100%, separate the upper- and lower-triangular portions of the matrix, which differ in how the sequence identity was calculated, specifically, which sequence was used as the “template” and which as the “query”, as the differing sequences have different lengths*.

Each of these five 454 pyrosequencing-determined sequence groups was analyzed for mutations, insertions, and deletions relative to the input plasmid sequence, as well as total number of reads and their redundancy (Table 2). For four of the plasmids ∼50,000 reads were obtained; for gVRC-H6_d74_, however, only about one fourth as many were obtained, which may relate to a lower efficiency of the primer used for gVRC-H6_d74_. In terms of redundancy, for three of the plasmids between one fifth and one half of the reads were identical to the input plasmid, whereas for VRC01 and gVRC-H6_d74_, only a small fraction (<1 and <10%) of the reads were identical to the input plasmid, a result of insertions in most of the sequences. Note that after error correction, 20–3254 more sequences became identical to the input antibodies (Table 2). Overall, for an antibody of typical length, ∼5-nucleotide mutations were observed between 454 pyrosequencing reads and corresponding input sequences. Error correction appeared to cause an increased count of mutation errors while decreasing insertion and deletion errors that produce stop codons and nonsense codons in protein translation. Currently used correction procedure was able to improve the identity of translated protein sequence to respective germline gene by an average of 14.1% (Figures S1C,D in Supplementary Material).

**Table 2:** **Statistical analysis of 454 pyrosequencing-induced errors for five plasmid antibodies**.

Antibody	Length (nt)	*N*_Seq_	*N*_Iden_	Unnormalized			Normalized (per 100 nt)		
				RMS_Mut_ (nt)	RMS_Ins_ (nt)	RMS_Del_ (nt)	RMS_Mut_ (nt)	RMS_Ins_ (nt)	RMS_Del_ (nt)
**NO ERROR CORRECTION**									
VRC01	363	47542	289	5.0	2.2	1.7	1.38	0.61	0.47
VRC03	390	53734	12309	4.6	4.1	0.9	1.18	1.05	0.23
VRC-PG04_cog_	369	43718	21281	5.0	1.7	0.5	1.36	0.46	0.14
gVRC-H3_d74_	381	53147	19843	6.3	1.9	1.1	1.65	0.50	0.29
gVRC-H6_d74_	399	13639	1013	6.4	2.8	1.6	1.60	0.70	0.40
**WITH ERROR CORRECTION**									
VRC01	363	47542	334	5.8	1.9	1.2	1.60	0.52	0.33
VRC03	390	53734	12948	4.7	4.0	0.9	1.21	1.03	0.23
VRC-PG04_cog_	369	43718	22021	5.1	1.6	0.4	1.38	0.43	0.11
gVRC-H3_d74_	381	53147	23097	6.5	1.7	0.9	1.71	0.45	0.24
gVRC-H6_d74_	399	13639	1033	6.6	2.7	1.5	1.65	0.68	0.38

*Columns include antibody name, nucleotide-sequence length of antibody heavy-chain variable domain, number of 454 pyrosequencing-determined heavy-chain variable-domain sequences for this antibody, number of sequences 100% identical to the sequenced antibody heavy-chain variable-domain, root-mean-square (RMS) fluctuation of 454 pyrosequencing-induced mutations, insertions, and deletions with respect to the input antibody sequence, and their values after normalization by a length of 100 nucleotides*.

*As shown in the percent sequence identity matrix in Table 1 and the divergence/identity plots in Figure 1, only five antibodies can be distinguished from others using a single sequence identity cutoff. After mapping the 454 pyrosequencing-determined heavy-chain variable-domain sequences onto 10 plasmid antibodies, a single cutoff of 75% was applied to extract sequences corresponding to VRC01, VRC03, VRC-PG04_cog_, gVRC-H3_d74_, and gVRC-H6_d74_, respectively*.

RMS_Mut_, RMS_Ins_, and RMS_Del_ were calculated using the formula:

${\text{RMS}}_{X}=\sqrt{\overset{N_{\text{Seq}}}{\underset{i}{\sum }}\frac{{\left(X_{i}-\bar{X}\right)}^{2}}{N_{\text{Seq}}}}$,

**X* denotes the type of sequencing error to be characterized, mutation (Mut), insertion (Ins), and deletion (Del), respectively; $\bar{X}$ denotes the averaged sequencing error; *N*_Seq_ denotes the total number of sequences within a given antibody group*.

*The RMS values were normalized using RMS_normalized_ = RMS_unnormalized_/lengh_unnormalized_ × 100 to take into account the difference in sequence length*.

We then examined the accuracy of bioinformatically selected representative sequences for these five antibody groups. Note that all these sequences have been subjected to a template-based error-correction procedure in the pipeline processing. A “divide-and-conquer” procedure (See Materials and Methods) was used for sequence calculation. Remarkably, the representative sequence was 100% identical to the “true” sequence used as input for 454 pyrosequencing for VRC-PG04_cog_, gVRC-H3_d74_, and gVRC-H6_d74_, while having one 1-nucleotide deletion and two 1-nucleotide insertions for VRC01 and VRC03, respectively. None had mutation errors. Such consensus-based sequence picking procedure may prove useful in the cases where a population of closely related sequences is observed on the divergence/identity plot, as indicated by a densely populated island.

### 454 pyrosequencing of donor 39 IGHV4 family and bioinformatics analysis of heavy chains

We next performed 454 pyrosequencing of PGT135–137-related heavy-chain transcripts from donor 39 PBMCs. mRNA from ∼5 million PBMCs was used for reverse transcription to produce template cDNA, and PCR was used to amplify IgG and IgM heavy-chain sequences from the IGHV4 family using forward primers that overlapped the end of the V gene leader sequence and the start of the V region and reverse primers covering the start of the constant domain (Table S1 in Supplementary Material).

Next-generation pyrosequencing provided 918,298 reads, which were processed with a bioinformatics pipeline that involved assignment of germline origin genes, 454 pyrosequencing-error correction, and extraction of CDR H3 regions for lineage assignment. Overall about 85.3% of the raw reads spanned over 400 nucleotides, covering the entire variable domain. After computational assignment of V, D, and J gene components, 142,842 sequences were assigned to IGHV4-39 germline family, accounting for ∼16% of the expressed VH4 antibodyome. Each sequence was subjected to an automatic error-correction scheme. For donor 39 heavy chains, the correction procedure improved the accuracy of protein translation, measured by protein sequence identity to inferred gemline gene, by an average of 20.4%. The results for pipeline processing of heavy-chain data set are listed in Figure S3 in Supplementary Material.

First, germline family analyses were performed using two standard methods – IMGT (Brochet et al., 2008) and IgBLAST (see text footnote 1; Table 3). These analyses assigned PGT135–137 gene origins to IGHV4-39 with two possible alleles (*03 or *07), to three potential D genes, and the J gene IGHJ5*02. An analysis of the third complementarity-determining region of the heavy chain (CDR H3) showed 80–90% sequence identity between PGT135–137, suggestive of a common lineage. The likely clonal origin of PGT135–137 indicates that they will all have the same V(D)J origin, with the different origin gene assignments by IMGT and IgBLAST likely due to their high divergence of ∼20% from ancestral gene.

**Table 3:** **Recombination origins of antibodies PGT135–137**.

Antibody	V gene (SeqID_togerm_)	D gene	J gene (SeqID_togerm_)	SeqID_toPGT135_
**HEAVY CHAIN (CDR H3 LENGTH = 18)**				
PGT135	IGHV4-39*07 (81.4%)	IGHD3-9*01	IGHJ5*02 (72.0%)	100.0%
PGT136	IGHV4-39*07 (82.1%)	IGHD2-8*02	IGHJ5*02 (78.0%)	83.0%
PGT137	IGHV4-39*03 (77.2%)	IGHD2-15*01	IGHJ5*02 (78.0%)	82.0%
**LIGHT CHAIN (CDR-L3 LENGTH = 9)**				
PGT135	IGKV3-15*01 (82.4%)	–	IGKJ1*01 (94.3%)	100.0%
PGT136	IGKV3-15*01 (86.4%)	–	IGKJ1*01 (97.1%)	84.4%
PGT137	IGKV3-15*01 (87.8%)	–	IGKJ1*01 (97.1%)	85.0%

*V, D, and J gene components of PGT135–137 were determined by two servers: IgBLAST (http://www.ncbi.nlm.nih.gov/igblast/) and IMGT/V-Quest (http://www.imgt.org/IMGT_vquest/vquest)*.

*SeqID_togerm_ is the nucleotide-sequence identity with respect to the germline gene*.

*SeqID_toPGT135_ is the nucleotide-sequence identity with respect to PGT135*.

**Figure 2:**
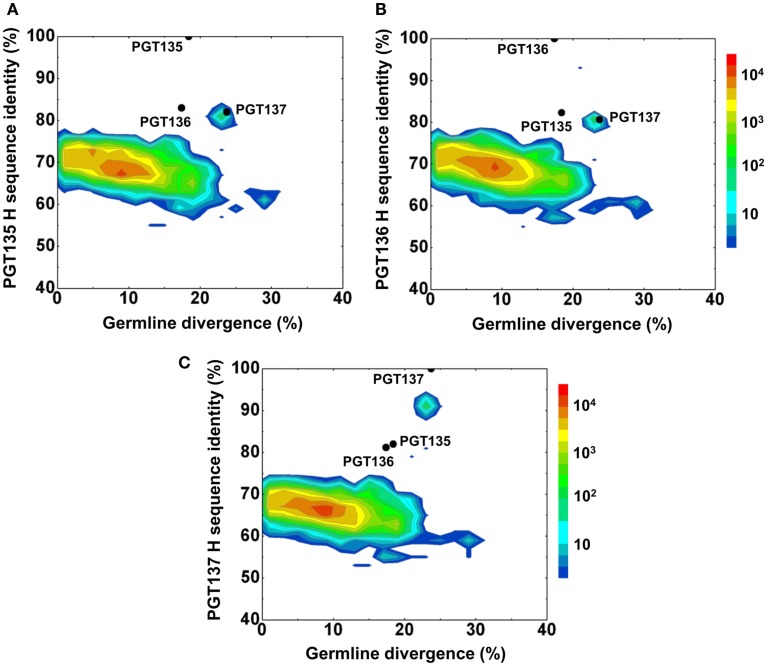
**Repertoire of donor 39 heavy-chain variable-domain sequences of IGHV4-39 origin determined by 454 pyrosequencing**. After processing by a standard bioinformatics pipeline (see Materials and Methods), 1,41,298 full-length, heavy-chain variable-domain sequences from IGHV4-39 germline family were obtained. These are plotted as a function of sequence identity to the heavy-chain variable domain of PGT135 **(A)**, PGT136 **(B)**, and PGT137 **(C)** and of sequence divergence from inferred IGHV4-39 germline allele. Color coding indicates the number of sequences. The 10%-identity gap indicates that the sequences within the upper island in 2C are somatic variants of PGT137 and not caused by sequencing errors.

Second, a divergence/identity analysis of 454 pyrosequencing-derived sequences assigned to IGHV4-39 origin was performed (Figure 2). The IGHV4-39-related sequences revealed a maximum divergence of 30.4% and an average divergence of 7.7% from germline. An island of sequences was observed at ∼90% identity to PGT137 with divergence of 20–25% from VH4-39, indicative of PGT137-related antibodies with similar evolutionary distance from the origin.

**Figure 3:**
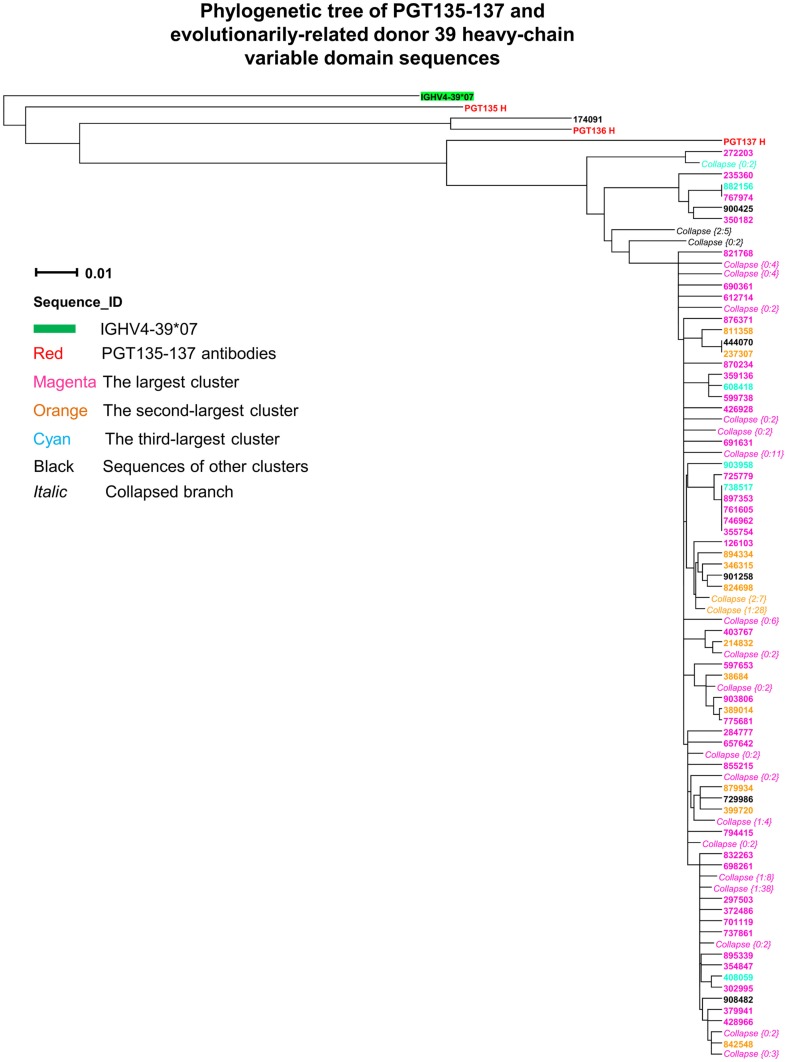
**Evolutionary similarity of PGT135–137 to donor 39 heavy-chain variable-domain sequences**. Germline-rooted maximum-likelihood tree of PGT135–137 and 202 sequences identified by the iterative intra-donor phylogenetic analysis of donor 39 heavy-chain variable domain sequences determined by 454 pyrosequencing. The iterative intra-donor phylogenetic analysis was based on an implementation of neighbor-joining (NJ) method. Collapsed branches are indicated by *Collapse {N: M}*, in which *N* is the branch depth (number of intermediate nodes) and *M* is the number of sequences within the branch. All sequences are on the PGT137 branch except for 174091, which is somatically related to PGT136.

Third, intra-donor phylogenetic analysis (see Materials and Methods) was applied to identify the somatic variants of PGT135–137 from the donor 39 heavy-chain sequencing data. In this analysis, a set of clonally related template antibodies is used to interrogate sequences from the same donor using phylogenetic analysis. Phylogenetic analysis, using a tree rooted by the inferred germline gene IGHV4-39*07, produced a ML dendrogram with 202 heavy-chain variable-domain sequences identified by their co-segregation with PGT135–137 (Figure 3). Most of the intra-donor-identified sequences clustered with PGT137, and one sequence clustered with PGT136.

Fourth, CDR H3 variation was analyzed for the 202 PGT135–137-related heavy-chain variable-domain sequences. One hundred seven were found to have identical CDR H3 sequences, as the same as the nucleotide-sequence consensus. With a maximum of five mutations from the consensus, the average CDR H3 variation was 1.2, indicative of a rather conserved signature of PGT135–137 lineage.

### PGT135–137 somatic heavy-chain populations and functional characterization

**Figure 4:**
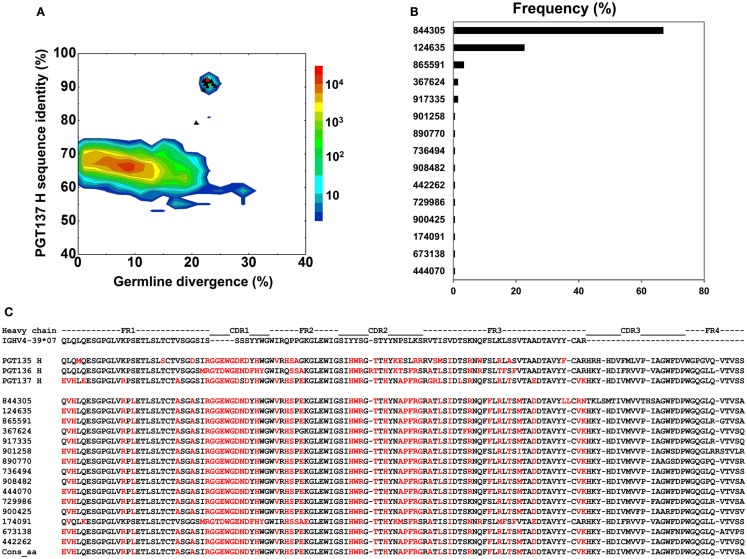
**Sequence selection for functional characterization of heavy chains from donor 39**. **(A)** Divergence/identity analysis of 15 heavy-chain variable-domain sequences obtained from the clustering analysis of 202 sequences identified by intra-donor phylogenetic analysis. Sequences of IGHV4-39 origin are plotted as a function of sequence identity to PGT137 heavy chain and sequence divergence from inferred germline allele, with 15 selected sequences shown as black triangles and their amino-acid consensus as red triangle. **(B)** Percent population of 15 clusters obtained using a sequence identity cutoff of 95%. Each cluster is indicated by its representative sequence. “Frequency” refers to the total number of sequences observed for each cluster. **(C)** Protein sequences of 15 cluster representatives and their amino-acid consensus. Sequences are aligned to the inferred germline gene, IGHV4-39*07. Framework regions (FR) and complementarity-determining regions (CDRs) are based on Kabat nomenclature. Amino acids mutated from the germline gene are shown in red.

To gain insight into the functional diversity of the antibodies identified by 454 pyrosequencing and bioinformatics methods, a clustering procedure was used to analyze the 202 identified heavy chains and to select representative sequences for further characterization. We used BLASTClust (Altschul et al., 1997) clustering function and an identity cutoff of 95% to sample the natural variation. We chose this cutoff to be greater than the ∼1.6% “false” sequence variation induced by 454 pyrosequencing errors (Table 2). A total of 15 clusters emerged. In the BLASTClust output, the first sequence of each cluster was selected to “represent” the cluster (Figure 4A) and were synthesized and reconstituted with the PGT137 light chain for functional assessment of HIV-1 neutralization, which was carried out on two viruses sensitive to PGT135–137 antibodies. Out of 15 tested heavy-chain variable domain sequences, when paired with PGT137 light chain, 11 reconstituted antibodies showed neutralization to different extents (Table 4).

**Table 4:** **Neutralization titers of 21 chimeric antibodies derived from 454 pyrosequencing of donor 39 against HIV-1 pseudoviruses from clade A and clade D**.

Seq. index	Nab name	Neutralization IC_50_ titers (μg/ml)			Neutralization IC_80_ titers (μg/ml)		
		RW020.2	UG024.2	MuLV	RW020.2	UG024.2	MuLV
**HEAVY-CHAIN VARIANT PAIRED WITH PGT135 LIGHT CHAIN**							
124635	gVRC-H1_d39_	0.005	0.021	>50	0.022	0.107	>50
865591	gVRC-H2_d39_	0.004	0.017	>48	0.016	0.08	>48
367624	gVRC-H3_d39_	0.009	0.253	>50	0.044	1.41	>50
917335	gVRC-H4_d39_	0.003	0.243	>50	0.015	3.41	>50
736494	gVRC-H5_d39_	0.003	0.458	>50	0.021	4.18	>50
442262	gVRC-H6_d39_	0.409	18.4	>50	12.8	>50	>50
729986	gVRC-H7_d39_	2.31	>50	>50	>50	>50	>50
900425	–	>50	>50	>50	>50	>50	>50
174091	gVRC-H8_d39_	>50	0.02	>50	>50	0.072	>50
673138	gVRC-H9_d39_	1.49	1.86	>50	23.6	11.5	>50
444070	gVRC-H10_d39_	0.008	0.027	>50	0.03	0.138	>50
ConsAA	gVRC-H11_d39_	0.003	0.016	>50	0.012	0.08	>50
**LIGHT-CHAIN VARIANTS PAIRED WITH PGT137 HEAVY CHAIN**							
107548	gVRC-L1_d39_	0.0007	0.008	>50	0.006	0.036	>50
219622	–	>50	>50	>50	>50	>50	>50
210137	–	>50	>50	>50	>50	>50	>50
215528	–	>50	>50	>50	>50	>50	>50
425756	gVRC-L2_d39_	<0.0006	0.007	>50	0.004	0.033	>50
121553	gVRC-L3_d39_	0.04	0.423	>50	0.245	2.16	>50
303540	gVRC-L4_d39_	0.075	1.11	>50	0.841	6.61	>50
378597	gVRC-L5_d39_	0.03	0.375	>50	0.167	1.26	>50
521298	–	>50	>50	>50	>50	>50	>50
537707	gVRC-L6_d39_	0.012	0.115	>50	0.057	0.436	>50
**WILD-TYPE PGT135–137 ANTIBODIES AND VRC01 ANTIBODY AS CONTROLS**							
PGT135	PGT135	>50	0.002	>50	>50	0.007	>50
PGT136	PGT136	>50	0.008	>50	>50	0.026	>50
PGT137	PGT137	0.001	0.007	>50	0.006	0.041	>50
VRC01	VRC01	0.157	0.207	>50	0.682	0.718	>50

*Columns include sequence index (for heavy chains, the amino-acid consensus is denoted by “ConsAA”; for controls, the antibody name is used as sequence index), neutralizing antibody name based on the nomenclature used in previous studies (Wu et al., 2011; Zhu et al., under review), neutralization IC_50_ and IC_80_ titers for viruses RW020.2 (HIV-1 clade A), UG024.2 (HIV-1 clade D) and MuLV (murine leukemia virus). Two sets of chimeric antibodies, 22 in total, were expressed by pairing the 12 heavy chains derived from 454 pyrosequencing and the PGT137 light chain, and pairing the 10 light chains derived from 454 pyrosequencing and the PGT137 heavy chain*.

*The wild-type mAbs PGT135–137 and wild-type VRC01 were included as controls*.

*MuLV stands for murine leukemia virus, which was included as a negative control*.

*“–” denotes expressed but non-neutralizing sequence after reconstituted with the PGT137-partner chain*.

The two largest clusters, with 136 and 46 sequences, respectively, accounted for ∼90% of the sequences (Figure 4B), while 10 of the 15 clusters contained only a single member. A consensus sequence (ConsAA), calculated from the alignment of 15 representative sequences (Figure 4C), was also synthesized. Notably, the reconstituted amino-acid consensus displayed neutralization almost on par with wild-type PGT137 (Table 4).

**Figure 5:**
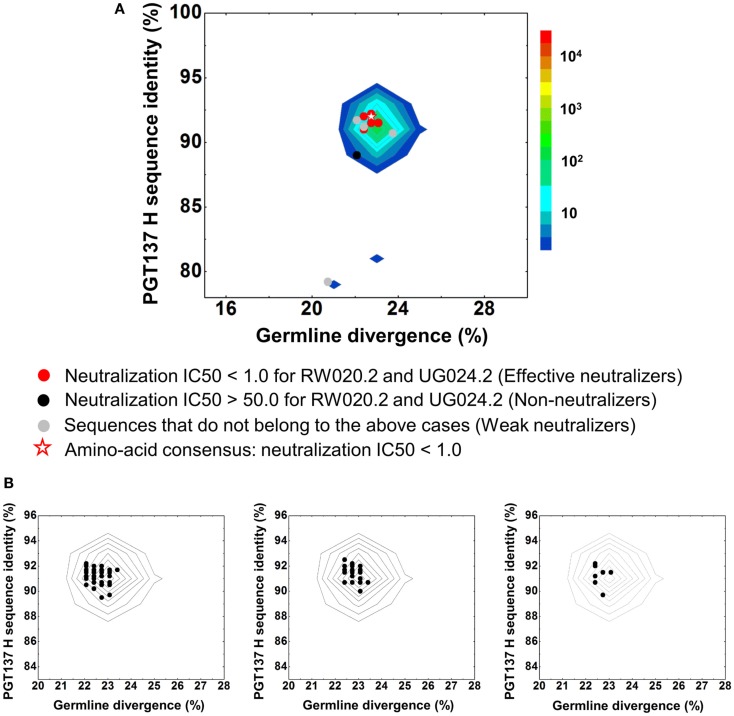
**Divergence/identity analysis of heavy-chain neutralization**. **(A)** The expressed heavy-chain sequences color-coded based on the neutralization potency of reconstituted antibodies, with IC50 <1.0 for both viruses shown in red (effective neutralizers), IC50 >50.0 for both viruses in black (non-neutralizers), and other cases in gray (weak neutralizers). The amino-acid consensus, when reconstituted with PGT137 light chain, neutralized both viruses with an IC50 <1.0 and is shown as a red hollow star. **(B)** The three largest clusters are displayed on the enlarged divergence/identity plot, with 136, 46, and 7 members, respectively.

Despite their apparent clonality, the clustering procedure reveals 15 clusters. The topology of the dendrogram produced from phylogenetic analysis indicates that these 15 clusters represent populations of somatically related antibodies evolving along distinct branches by standard mechanisms of hypermutation (Figure 3). We analyzed these 15 somatic populations for prevalence of mutations, insertions, and deletions (Table S2 in Supplementary Material). Note that the representative sequence of cluster 1 (#844305) contained two insertions in the CDR H3 region which were not seen in other members of the cluster, suggesting that these insertions might be sequencing errors. Indeed, this heavy chain could not be expressed when reconstituted with PGT137 light chain. We also analyzed each of these populations by divergence/identity plot (Figure 5). Overall, sequences chosen to represent the 15 somatic populations showed diverse neutralization characteristics (Table S2 in Supplementary Material). Some antibodies, for example from clusters 2, 3, 14, and 15 (gVRC-H1_d39_, gVRC-H2_d39_, gVRC-H9_d39_, and gVRC-H10_d39_), neutralized clade A – RW020.2 and clade D – UG024.2 with roughly equal potency. Some antibodies, for example from clusters 4, 5, 8, and 10 (gVRC-H3-H6_d39_), neutralized clade A-RW020.2 25-150-fold more potently than clade D. While the antibody from cluster 13 (gVRC-H8_d39_) neutralized clade D – UG024.2 with at least 100-fold greater potency than clade A. These results provide an example for how somatically related antibodies can significantly differ in their neutralization specificities. This begins to provide insight into how populations of somatically related antibodies can engender neutralization breadth significantly different than any individual member.

### 454 pyrosequencing of donor 39 IGKV3 family and bioinformatics analysis of light chains

We next performed 454 pyrosequencing of PGT135–137-related light-chain transcripts from donor 39 PBMCs. mRNA from ∼5 million PBMCs was used for reverse transcription to produce template cDNA, and PCR was used to amplify light-chain sequences from the IGKV3 family.

The 454 pyrosequencing provided 971,165 reads, which were then processed using a pipeline adapted for κ-chain analysis. For donor 39, about 83.3% of the raw reads were 400 nt or longer, effectively covering the light-chain variable domain. After V and J gene assignment, 91,951 sequences were determined to belong to IGKV3-15 germline family, accounting for 10% of the light chain reads obtained. After error correction, the accuracy of protein translation measured by the protein sequence identity to inferred gemline gene was improved by an average of 16.5%. The results for pipeline processing of light-chain data set are listed in Figure S4 in Supplementary Material.

First, the recombination origins of PGT135–137 light chains were analyzed (Table 3). PGT135–137 light chains were assigned to the same germline V gene allele, IGKV3-15*01, recombined with the same J gene, IGKJ1*01, supporting the notion that the discrepancy in heavy-chain germline assignment was likely an artifact caused by their high divergence.

**Figure 6:**
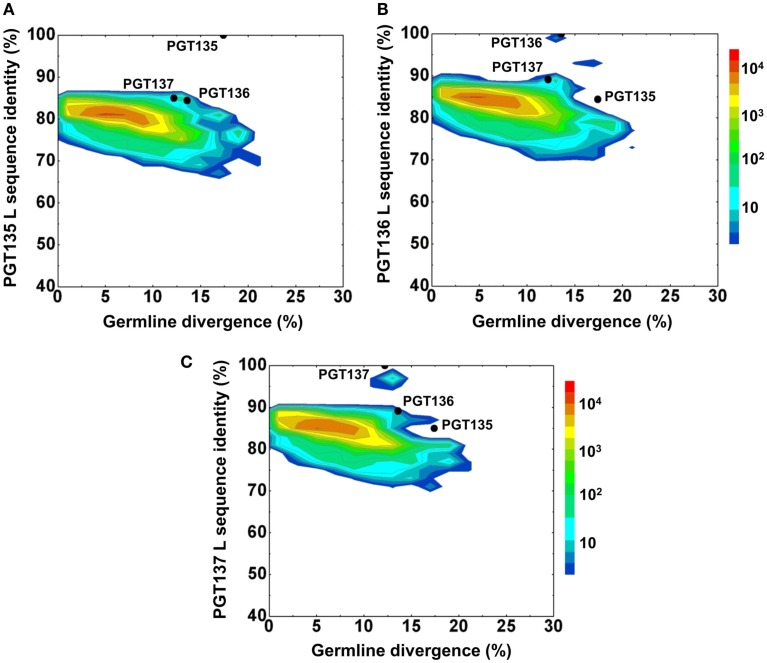
**Repertoire of donor 39 light-chain variable-domain sequences of IGKV3-15 origin determined by 454 pyrosequencing**. After processed by a standard bioinformatics pipeline, 87,229 full-length, light-chain variable-domain sequences from IGKV3-15 germline family are plotted as a function of sequence identity to the light-chain variable-domain of PGT135 **(A)**, PGT136 **(B)**, and PGT137 **(C)** and of sequence divergence from inferred IGKV3-15 germline allele. Color coding indicates the number of sequences.

Second, the divergence/identity analysis of 454 pyrosequencing-derived sequences assigned to the IGKV3-15*01 origin was performed (Figure 6). The IGKV3-15*01-related sequences revealed a maximum divergence of 20.9% and an average divergence of 6.3% from germline. Distinct sequence islands were observed at ∼100% identity to PGT136 and 95% identity to PGT137 – both with divergence of 10–15% from IGKV3-15*01. No distinct sequence island was observed that was closely related to PGT135.

**Figure 7:**
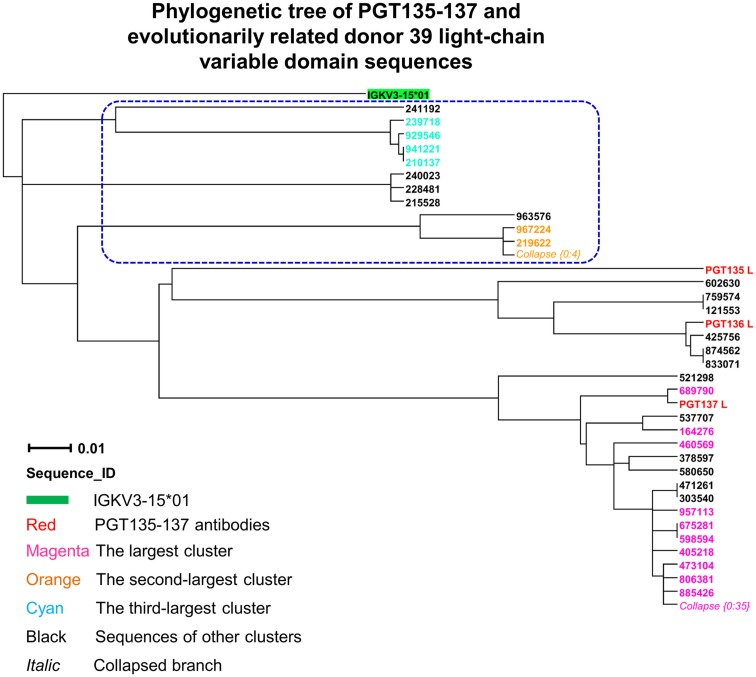
**Evolutionary similarity of PGT135–137 to donor 39 light-chain variable-domain sequences**. Germline-rooted maximum-likelihood tree of PGT135–137 and 72 sequences identified by the iterative intra-donor phylogenetic analysis of donor 39 light-chain variable-domain sequences determined by 454 pyrosequencing. The iterative intra-donor phylogenetic analysis was based on an implementation of neighbor-joining (NJ) method. Collapsed branches are indicated by *Collapse {N: M}*, as in Figure 3. Sequences that are immediately outside the maximum-likelihood-defined PGT135–137 subtree are circled in blue dashed-line.

Third, to identify light-chain somatic variants, we performed intra-donor phylogenetic analysis that combined an iterative NJ procedure for the high-throughput screening of sequencing data, and a ML calculation to confirm the NJ analysis and to provide the final dendrogram (see Materials and Methods). Two methods were usually in agreement, e.g., for donor 39 heavy chains, but differed here. The NJ-based analysis yielded 72 sequences within the PGT135–137 subtree, whereas the subsequent ML-based analysis retained 57 of the 72 sequences within the PGT135–137 subtree (Figure 7), providing an example for functional characterization of similar but somatically unrelated sequences.

### PGT135–137 somatic light-chain populations and functional characterization

**Figure 8:**
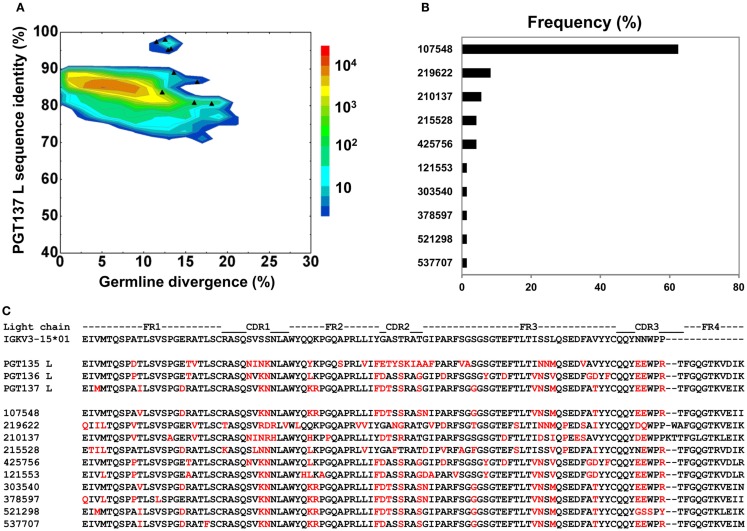
**Sequence selection for functional characterization of light chains from donor 39**. **(A)** Divergence/identity analysis of 10 light-chain variable-domain sequences obtained from the clustering analysis of 72 sequences identified by intra-donor phylogenetic analysis. Sequences of IGKV3-15*01 origin are plotted as a function of sequence identity to PGT137 light chain and sequence divergence from inferred germline allele, with 10 selected sequences shown as black triangles. **(B)** Percent population of 10 clusters obtained using a sequence identity cutoff of 95%. Each cluster is indicated by its representative sequence. **(C)** Protein sequences of 10 cluster representatives. Sequences are aligned to the inferred germline gene, IGKV3-15*01. Framework regions (FR) and complementarity-determining regions (CDRs) are based on Kabat nomenclature. Amino acids mutated from the germline gene are shown in red.

By using the same 95% clustering procedure as for heavy chains, 14 light-chain clusters were identified from the phylogenetic tree. Representative sequences were selected, also as described for heavy chains, from the first 10 clusters for functional characterization (Figure 8A). We analyzed these 10 clusters for prevalence of mutations, insertions, and deletions (Table S3 in Supplementary Material). The largest cluster, lying within the population of PGT137-like sequences, contained 45 sequences or 63% of the subtree sequences (Figure 8B). All selected light-chain sequences possessed CDR L3s of the same length except for the sequences selected from the clusters 2 and 3 (Figure 8C). Out of 10 tested light-chain variable domain sequences, when reconstituted with the PGT137 heavy chain, six antibodies – representing six sequence clusters – showed neutralization of two HIV-1 strains from clade A and clade D. Notably, two of the light chains (gVRC-L1_d39_ and gVRC-L2_d39_) showed neutralization breadth slightly better than PGT135–137, and the light-chain variants neutralized clade A about 10-fold more effectively than the clade D (Table 4).

**Figure 9:**
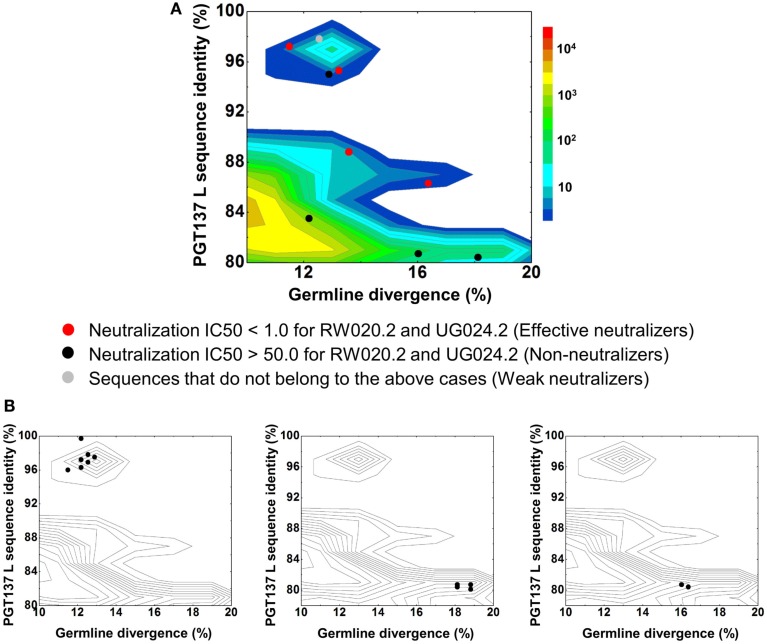
**Divergence/identity analysis of light-chain neutralization**. **(A)** The expressed light-chain sequences color-coded based on the neutralization potency of reconstituted antibodies, with IC50 <1.0 for both viruses shown in red (effective neutralizers), IC50 >50.0 for both viruses in black (non-neutralizers), and other cases in gray (weak neutralizers). **(B)** The three largest clusters are displayed on the enlarged divergence/identity plot, with 45, 6, and 4 members, respectively.

In contrast to the 454 pyrosequencing-identified heavy chains, the six neutralizing light-chain clusters were not localized to a single divergence/identity island (Figure 9). Indeed, neutralization was observed with clusters from at least three diverse locations on the divergence/identity plot. Nevertheless, the topology of the light-chain phylogenetic analysis indicates that these six clusters represent populations of somatically related antibodies (Figure 7).

## Discussion

Recently, select antibodies with the ability to neutralize diverse strains of HIV-1 have been identified in HIV-1 infected donors (Walker et al., 2009, 2011; Corti et al., 2010; Wu et al., 2010, 2011; Scheid et al., 2011). Like PGT135–137, antibodies from these donors often appear to be clonally related, to possess similar neutralization characteristics, and to cluster in a localized island (or islands) on identity/diversity plots. These islands observed in 454 pyrosequencing-derived analyses are often nearby but rarely overlap the few antibodies experimentally isolated from the same individual (even if they start with samples of exactly the same time point, as we have done here with donor 39). The differences between antibodies identified from sorting of memory B cells or by 454 pyrosequencing of B cell transcripts suggest that the experimental approaches may capture or sample different B cell population. In addition to exploring differences in phenotype of antibody identified by the two methods, we also explored differences related to the quantity of identified antibody. In particular, we ask whether the less-sparse view of the antibody repertoire provided by next-generation sequencing and systems-level bioinformatics might provide insight into the diversity of the antibody response.

With the heavy chains of PGT135–137, select sequences representing 15 distinct populations, showed dramatically different neutralization characteristics toward clade A and D viruses when reconstituted with the same light chain from PGT137. With the light chains of PGT135–137, select sequences representing 10 distinct populations were not localized to a discrete sequence island, indicating substantial differences in identity and diversity (Figure 8). Thus, even though these antibodies are somatically related, both their neutralization and sequence characteristics can diverge substantially (Table 4). These results demonstrate the utility of next-generation sequencing, which provides a more comprehensive sampling of sequences, and of systems-level bioinformatics approaches, which enable these data to be mined effectively. Overall, data-intensive methods may be generally required to obtain true insight into questions of biological diversity such as the humoral immune response.

Prior next-generation sequencing and bioinformatics analyses have revealed the extraordinary genetic diversity of HIV-1 (Eriksson et al., 2008; Archer et al., 2009; Tsibris et al., 2009; Fischer et al., 2010). These same methods are now beginning to reveal the extraordinary diversity of antibodies generated in response to HIV-1 infection (Wu et al., 2011). Although this response appears to provide little benefit to the HIV-1-infected host (Poignard et al., 1999), if similar responses could be generated through vaccination, then in principle effective protection could be achieved in the setting of initial infection (Burton, 2002; Burton et al., 2004, 2005). The populations of antibodies we identify here may provide broader protection than a monoclonal member of the group. Furthermore, responses to infection or vaccination would be expected to generate diverse populations of antibodies, as we have shown here. Thus, population diversity, even within a single antibody clone or lineage, is likely to have a substantial impact on the effectiveness of the immune response.

## Data Deposition

Next-generation sequencing data from donor 39 (heavy and light chains) and also for the 10 plasmid control have been deposited in the National Center for Biotechnology Information Short Reads Archives (SRA) under accession no. SRA055820. Information deposited with GenBank includes the heavy- and light-chain variable region sequences of genomically identified neutralizers: 10 heavy chains, gVRC-H1-10_d39_ (JX313021-30), amino-acid consensus heavy-chain gVRC-H11_d39_ (JX444560), and 6 light chains, gVRC-L1-6_d39_ (JX313030-36).

## Conflict of Interest Statement

The authors declare that the research was conducted in the absence of any commercial or financial relationships that could be construed as a potential conflict of interest.

## Supplementary Material

The Supplementary Material for this article can be found on line at http://www.frontiersin.org/Virology/10.3389/fmicb.2012.00315/abstract

Supplementary Figure S1 **Pipeline processing of heavy-chain sequences of 10 plasmid antibodies determined by 454 pyrosequencing**.

Supplementary Figure S2 **Pyrosequencing-induced sequence variation for 10 plasmid antibodies after being processed by an error-correction procedure**.

Supplementary Figure S3 **Pipeline processing of donor 39 heavy-chain sequences determined by 454 pyrosequencing**.

Supplementary Figure S4 **Pipeline processing of donor 39 light-chain sequences determined by 454 pyrosequencing**.

Supplementary Table S1 **PCR primers and DNA polymerase systems used to prepare samples for 454 pyrosequencing**.

Supplementary Table S2 **Neutralization of reconstituted antibodies by pairing clustering-selected heavy-chain sequences from 454 pyrosequencing with PGT137 light chain**.

Supplementary Table S3 **Neutralization of reconstituted antibodies by pairing clustering-selected light-chain sequences from 454 pyrosequencing with PGT137 heavy chain**.

## Footnotes

^1^http://www.ncbi.nlm.nih.gov/igblast/

^2^http://evolution.genetics.washington.edu/phylip/doc/dnamlk.html

^3^http://evolution.genetics.washington.edu/phylip.html
