# Supplementary material for: Somatic Populations of PGT135–137 HIV-1-Neutralizing Antibodies Identified by 454 Pyrosequencing and Bioinformatics
Source: Front Microbiol. 2012 Sep 11;3:315. doi: 10.3389/fmicb.2012.00315 (PMC3441199; doi:10.3389/fmicb.2012.00315)
Supplement: Supplementary Figure S1 — Pipeline processing of heavy-chain sequences of 10 plasmid antibodies determined by 454 pyrosequencing. [file 31400_Kwong_Presentation1.PDF]

Supplementary Material

Somatic populations of PGT135-137 HIV-1-neutralizing antibodies identified by 454 pyrosequencing and bioinformatics

Running title: Somatic populations of HIV-1-neutralizing antibodies

Jiang Zhu[1],
Sijy O'Dell[1#], Gilad Ofek[1#], Marie Pancera[1#], Xueling Wu[1#], Baoshan Zhang[1#], Zhenhai Zhang[2#],
NISC Comparative Sequencing Program[3], James C. Mullikin[3],
Melissa Simek[4], Dennis R. Burton[5,6], Wayne C. Koff[4],
Lawrence Shapiro[1,2], John R. Mascola[1] and Peter D. Kwong[1*]

[1] Vaccine Research Center, National Institute of Allergy and Infectious Diseases, National Institutes of Health, Bethesda, MD 20892, USA.
[2] Department of Biochemistry and Molecular Biophysics, Columbia University, New York, NY 10032, USA.
[3] NIH Intramural Sequencing Center (NISC), National Human Genome Research Institute, National Institutes of Health, Bethesda, Maryland 20892, USA
[4] International AIDS Vaccine Initiative (IAVI), New York, NY 10038, USA.
[5] Department of Immunology and Microbial Science and IAVI Neutralizing Antibody Center, The Scripps Research Institute, La Jolla, California 92037, USA.
[6] Ragon Institute of MGH, MIT, and Harvard, Cambridge, Massachusetts 02129, USA.

[#]Equal contribution

[*]Correspondence should be addressed to P.D.K. Vaccine Research Center, NIAID/NIH
40 Convent Drive; Building 40, Room 4508, Bethesda, MD 20892; Phone: (301) 594-8685; Fax: (301) 480-2658, E-mail: pdkwong@nih.gov.

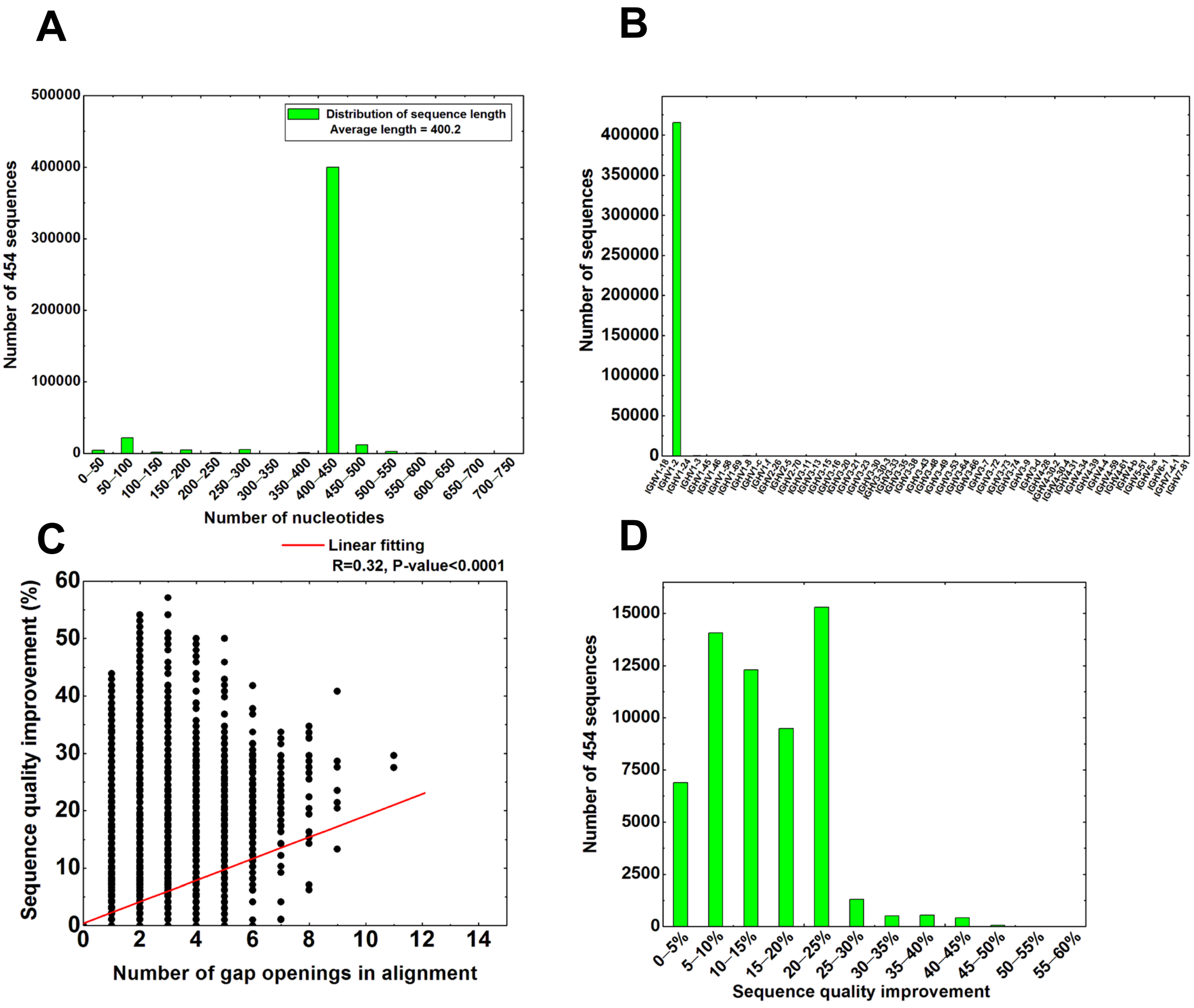

Figure S1. Pipeline processing of heavy chain sequences of 10 plasmid antibodies determined by 454 pyrosequencing. (A) Read length distribution (step 1). Of 456,735 reads, 415,748 (or 91.0%) were 400 nt or longer; (B) Germline family distribution (step 2). In this step, given a sequence read, V(D)J components were determined using a local implementation of IgBLAST. Sequences with an E-value of 1.0E-3 or greater for V gene assignment were removed. Note that 421,541 sequences (or 93.3%) were correctly assigned to VH1-2, the germline gene of these 10 plasmid antibodies; (C) Correlation between number of gaps in alignment and sequence quality improvement, which is measured by the increase of amino-acid sequence identity to germline V gene after error correction (step 3). The correlation coefficient was 0.32 and P-value was less than 0.0001; (D) Sequence quality improvement distribution (step 3), with an average improvement of 14.1%. In this step, a template-based correction procedure was applied to correct homopolymer errors in V, D and J genes. Note that D and J genes were subjected to this correction procedure only when their E-values were significant (1.0E-3 or lower). The identities to the heavy chains of 10 plasmid antibodies were calculated in step 4, and the exact boundaries for variable domain and CDR H3 region were calculated in step 5.

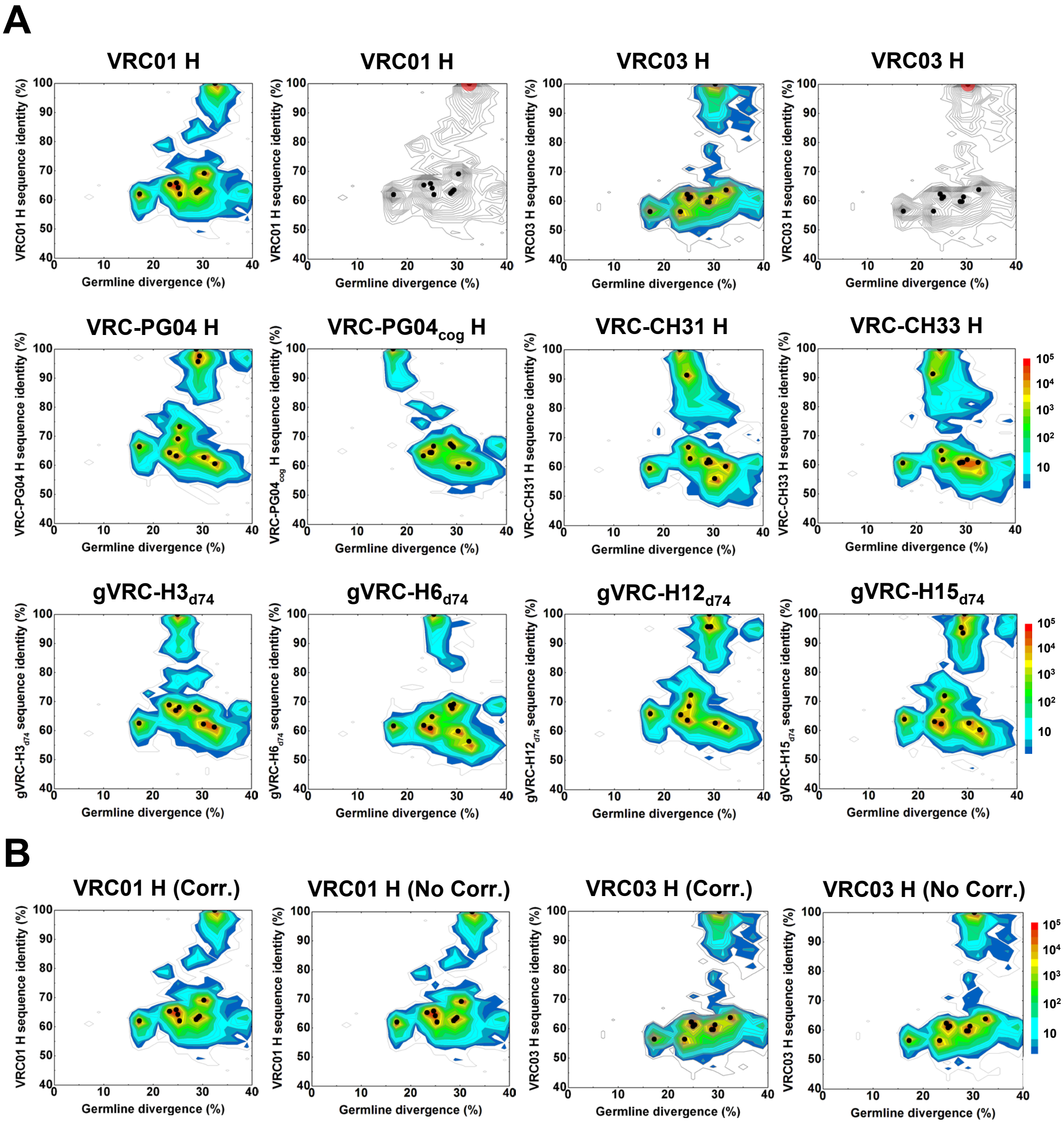

Figure S2. 454-pyrosequencing-induced sequence variation for 10 plasmid antibody data set processed with error correction. Tested antibodies include VRC01, VRC03, VRC-PG04, VRC-CH31, VRC-CH33, a codon-optimized version of inferred, reverted unmutated ancestor of VRC-PG04 (termed VRC-PG04$_{cog}$), gVRC-H3$_{d74}$, gVRC-H6$_{d74}$, gVRC-H12$_{d74}$ and gVRC-H15$_{d74}$. (A) Divergence/identity analysis of 454-pysequencing-determined heavy chain variable domain sequences. For VRC01 and VRC03, the estimated mutational error range is indicated by red shade around the input antibody on the gray contour plot. (B) Comparison of divergence/identity plots with and without error correction for VRC01 and VRC03. Since divergence and identity were calculated at the nucleotide level, the error correction had little effect on the sequence distribution. Color coding indicates the number of sequences.

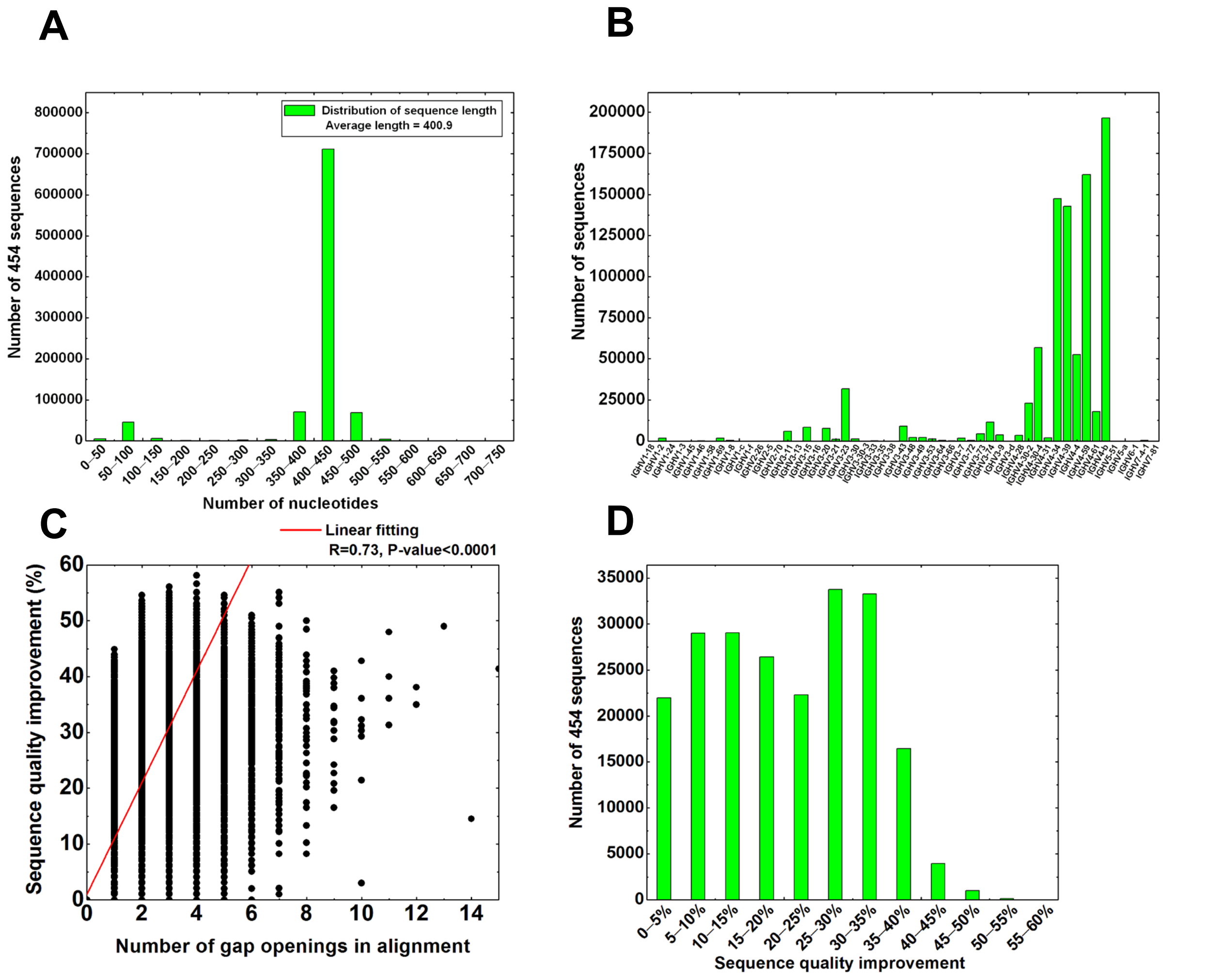

Figure S3. Pipeline processing of donor 39 heavy chain sequences determined by 454 pyrosequencing. (A) Read length distribution (step 1). Of 918,298 reads, 784,196 (or 85.3%) were 400 nt or longer; (B) Germline family distribution (step 2). In this step, given a sequence read, V(D)J components were determined using a local implementation of IgBLAST. Sequences with an E-value of 1.0E-3 or greater for V gene assignment were removed. Note that 142,842 sequences were of VH4-39 origin, the germline gene of PGT135-137 heavy chains; (C) Correlation between number of gaps in alignment and sequence quality improvement, which is measured by the increase of amino-acid sequence identity to germline V gene after error correction (step 3). The correlation coefficient was 0.73 and P-value was less than 0.0001; (D) Sequence quality improvement distribution (step 3), with an average improvement of 20.4%. In this step, a template-based correction procedure was applied to correct homopolymer errors in V, D and J genes. Note that D and J genes were subjected to this correction procedure only when their E-values were significant (1.0E-3 or lower). The identities to PGT135-137 heavy chains were calculated in step 4, and the exact boundaries for variable domain and CDR H3 region were calculated in step 5.

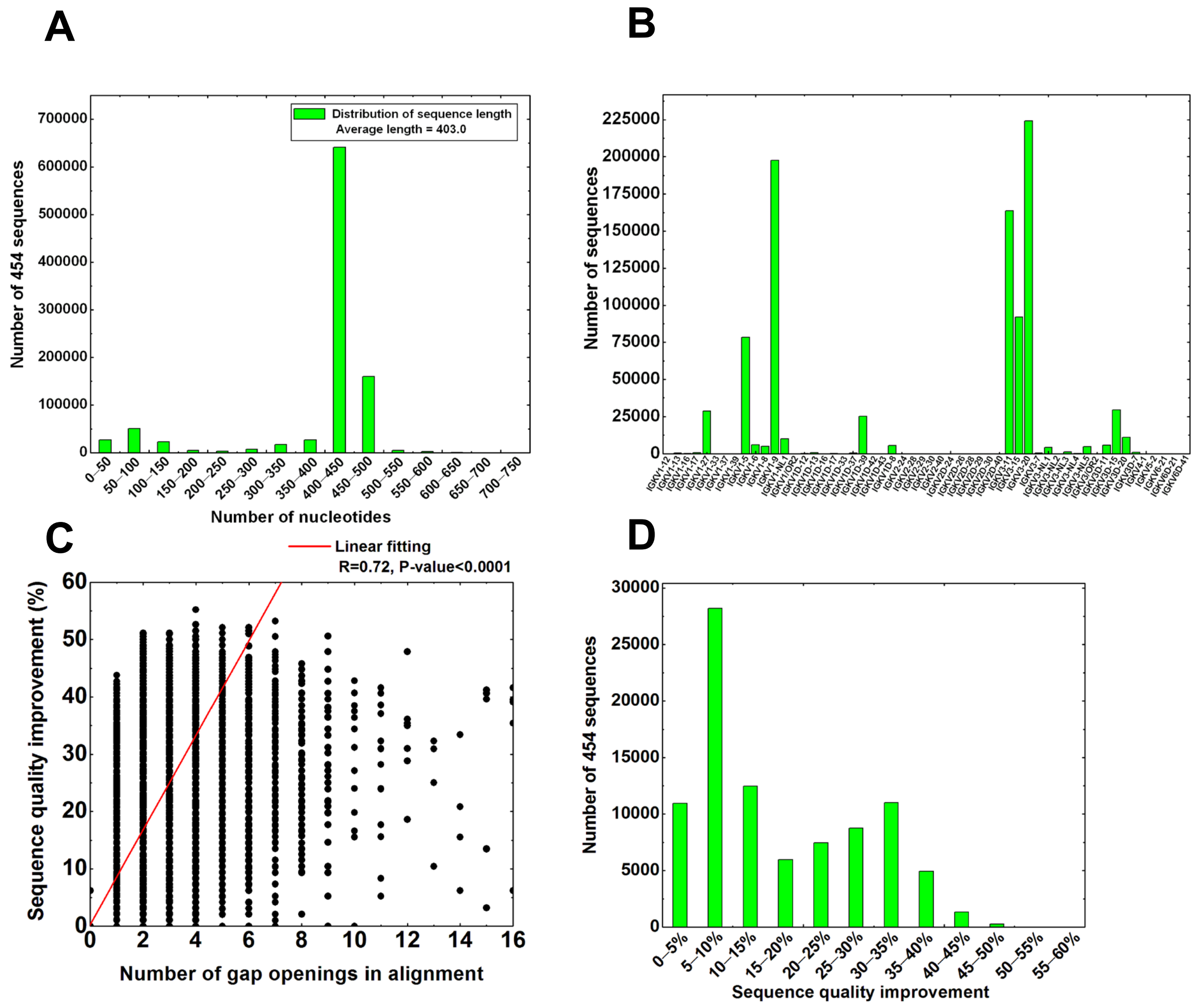

Figure S4. Pipeline processing of donor 39 light chain sequences determined by 454 pyrosequencing. (A) Read length distribution (step 1). Of 971,165 reads, 809,397 (or 83.3%) were 400 nt or longer; (B) Germline family distribution (step 2). In this step, given a sequence read, V and J components were determined using a local implementation of IgBLAST. Sequences with an E-value of 1.0E-3 or greater for V gene assignment were removed. Note that 91,951 sequences were of IGKV3-15 origin, the germline gene of PGT135-137 light chains; (C) Correlation between number of gaps in alignment and sequence quality improvement, which is measured by the increase of amino-acid sequence identity to germline V gene after error correction (step 3). The correlation coefficient was 0.72 and P-value was less than 0.0001; (D) Sequence quality improvement distribution (step 3), with an average improvement of 16.5%. In this step, a template-based correction procedure was applied to correct homopolymer errors in V and J genes. Note that J gene was subjected to this correction procedure only when E-value of its assignment was significant (1.0E-3 or lower). The identities to PGT135-137 light chains were calculated in step 4, and the exact boundaries for variable domain and CDR L3 region were calculated in step 5.

Table S1 | PCR primers used to prepare samples for 454 pyrosequencing.

10 antibody heavy chain plasmids PCR:

**Primer**	**Primer sequence (5' → 3')**
Forward H1 primers	
XLR-A_5'L-VH1	CCATCTCATCCCTGCGTGTCTCCGACTCAG ACAGGTGCCCACTCCCAGGTGCAG
XLR-A_5'L-VH1#2	CCATCTCATCCCTGCGTGTCTCCGACTCAG GCAGCCACAGGTGCCCACTCC
XLR-A_5'L-VH1-24	CCATCTCATCCCTGCGTGTCTCCGACTCAG CAGCAGCTACAGGCACCCACGC
XLR-A_5'L-VH1-69	CCATCTCATCCCTGCGTGTCTCCGACTCAG GGCAGCAGCTACAGGTGTCCAGTCC
Reverse primers	
XLR-B_3CγCH1#2	CCTATCCCCTGTGTGCCTTGGCAGTCTCAG GGGGAAGACCGATGGGCCCTTGGT
XLR-B_3CμCH1	CCTATCCCCTGTGTGCCTTGGCAGTCTCAG GGGAATTCTCACAGGAGACGA

Donor 39 PCR:

**Primer**	**Primer sequence (5' → 3')**
Forward VH4 primers	
XLR-A_5'L-VH4/6	CCATCTCATCCCTGCGTGTCTCCGACTCAG CCCAGATGGGTCCTGTCCCAGGTGCAG
XLR-A_5'L-VH3/4 #1	CCATCTCATCCCTGCGTGTCTCCGACTCAG GTGGCAGCTCCCAGATGGGTCCTGTC
XLR-A_5'L-VH3/4 #3	CCATCTCATCCCTGCGTGTCTCCGACTCAG GTTGCAGTTTTAAAAGGTGTCCAGTG
Reverse primers	
XLR-B_3CγCH1	CCTATCCCCTGTGTGCCTTGGCAGTCTCAG GGGGAAGACCGATGGGCCCTTGGTGG
XLR-B_3CμCH1	CCTATCCCCTGTGTGCCTTGGCAGTCTCAG GGGAATTCTCACAGGAGACGA
Forward kappa chain primer	
XLR-A_5'L-VK3	CCATCTCATCCCTGCGTGTCTCCGACTCAG CTCTTCCTCCTGCTACTCTGGCTCCCAG
Reverse kappa chain primer	
XLR-B_3'CK1	CCTATCCCCTGTGTGCCTTGGCAGTCTCAG CAGCAGGCACACAACAGAGGCAGTTCC

A germline gene analysis was carried out using IgBLAST (http://www.ncbi.nlm.nih.gov/igblast/) to determine appropriate primes for PGT135-137. The H1 primers developed in our previous study (Wu et al., 2011) were used here for 10 plasmid antibodies.

Table S2. Neutralization of reconstituted antibodies by pairing clustering-selected heavy chain sequences from 454 pyrosequencing with PGT137 light chain

		Neutralization IC_{50} titers (μg/ml)						
Cluster	$Index_{HC}$	RW020.2 (clade A)	UG024.2 (clade D)	MuLV (non-HIV)	RMS_{mut}	RMS_{Ins}	RMS_{Del}	Distance (M/I/D)
1 (136)	844305	—	—	—	2.1	1.1	0.9	0/9/0
2 (46)	124635	0.005	0.021	>50	2.3	1.4	0.9	0/0/0
3 (7)	865591	0.004	0.017	>48	4.5	5.0	0.9	6/0/0
4 (2)	367624	0.009	0.253	>50				
5 (1)	917335	0.003	0.243	>50				
6 (1)	901258	—	—	—				
7 (1)	890770	—	—	—				
8 (1)	736494	0.003	0.458	>50				
9 (1)	908482	—	—	—				
10 (1)	442262	0.409	18.4	>50				
11 (1)	729986	2.31	>50	>50				
12 (1)	900425	>50	>50	>50				
13 (1)	174091	>50	0.02	>50				
14 (1)	673138	1.49	1.86	>50				
15 (1)	444070	0.008	0.027	>50				
Consensus	ConsAA	0.003	0.016	>50				

Listed items include the heavy chain cluster index (and number of sequences within the cluster), sequence index of the selected heavy chain, neutralization IC_{50} titers for two HIV-1 Env-pseudoviruses from clade A and clade D, root-mean-square (RMS) fluctuation of the mutations (RMS_{Mut}), insertions (RMS_{Ins}) and deletions (RMS_{Del}) with respect to the consensus sequence within the cluster, and the 'distance' between the picked sequence and the consensus measured by number of mutations, insertions and deletions (M/I/D). Note that the sequence variation within a cluster was only calculated for clusters with more than 3 sequences.

'—' denotes no expression here.

Table S3. Neutralization of reconstituted antibodies by pairing clustering-selected light chain sequences from 454 pyrosequencing with PGT137 heavy chain

		Neutralization IC_{50} titers (μg/ml)						
Cluster	$Index_{LC}$	RW020.2 (clade A)	UG024.2 (clade D)	MuLV (non-HIV)	RMS_{mut}	RMS_{Ins}	RMS_{Del}	Distance (M/I/D)
1 (45)	107548	0.0007	0.008	>50	2.1	0.0	0.2	0/0/0
2 (6)	219622	>50	>50	>50	1.0	0.0	0.4	1/0/0
3 (4)	210137	>50	>50	>50	1.3	0.0	0.0	0/0/0
4 (3)	215528	>50	>50	>50				
5 (3)	425756	<0.0006	0.007	>50				
6 (2)	121553	0.04	0.423	>50				
7 (2)	303540	0.075	1.11	>50				
8 (1)	378597	0.03	0.375	>50				
9 (1)	521298	>50	>50	>50				
10 (1)	537707	0.012	0.115	>50				

Listed items include the light chain cluster index (and number of sequences within the cluster), sequence index of the selected light chain, neutralization IC_{50} titers for two HIV-1 Env-pseudoviruses from clade A and clade D, root-mean-square (RMS) fluctuation of the mutations (RMS_{Mut}), insertions (RMS_{Ins}) and deletions (RMS_{Del}) with respect to the consensus sequence within the cluster, and the 'distance' between the picked sequence and the consensus measured by number of mutations, insertions and deletions (M/I/D). Note that the sequence variation within a cluster was only calculated for clusters with more than 3 sequences.
Clusters 2 and 3 correspond to the sequences outside the PGT135-137 branch in the intra-donor phylogenetic tree in FIGURE 7.
